# Atrial secondary mitral regurgitation: prevalence, characteristics, management, and long-term outcomes

**DOI:** 10.1186/s44156-023-00015-y

**Published:** 2023-03-08

**Authors:** Sam Straw, Ankit Gupta, Kerryanne Johnson, Charlotte A. Cole, Kinan Kneizeh, John Gierula, Mark T. Kearney, Christopher J. Malkin, Maria F. Paton, Klaus K. Witte, Dominik Schlosshan

**Affiliations:** 1 University of Leeds, Leeds, UK; 2 Leeds Teaching Hospitals NHS Trust, Leeds, UK; 3 Nelson Hospital, Nelson, New Zealand; 4 University Hospital Aachen, RWTH, Aachen, Germany

**Keywords:** Valvular heart disease, Heart failure, Mitral regurgitation, Secondary mitral regurgitation, Atrial fibrillation

## Abstract

**Background:**

The prevalence, clinical characteristics, management and long-term outcomes of patients with atrial secondary mitral regurgitation (ASMR) are not well described.

**Methods:**

We performed a retrospective, observational study of consecutive patients with grade III/IV MR determined by transthoracic echocardiography. The aetiology of MR was grouped as being either primary (due to degenerative mitral valve disease), ventricular SMR (VSMR: due to left ventricular dilatation/dysfunction), ASMR (due to LA dilatation), or other.

**Results:**

A total of 388 individuals were identified who had grade III/IV MR; of whom 37 (9.5%) had ASMR, 113 (29.1%) had VSMR, 193 had primary MR (49.7%), and 45 (11.6%) were classified as having other causes. Compared to MR of other subtypes, patients with ASMR were on average older (median age 82 [74–87] years, *p* < 0.001), were more likely to be female (67.6%, *p* = 0.004) and usually had atrial fibrillation (83.8%, *p* = 0.001). All-cause mortality was highest in patients with ASMR (*p* < 0.001), but similar to that in patients with VSMR once adjusted for age and sex (hazard ratio [HR] 0.81, 95% confidence interval [CI] 0.52–1.25). Hospitalisation for worsening heart failure was more commonly observed in those with ASMR or VSMR (*p* < 0.001) although was similar between these groups when age and sex were accounted for (HR 0.74, 95% CI 0.34–1.58). For patients with ASMR, the only variables associated with outcomes were age and co-morbidities.

**Conclusions:**

ASMR is a prevalent and distinct disease process associated with a poor prognosis, with much of this related to older age and co-morbidities.

**Graphical Abstract:**

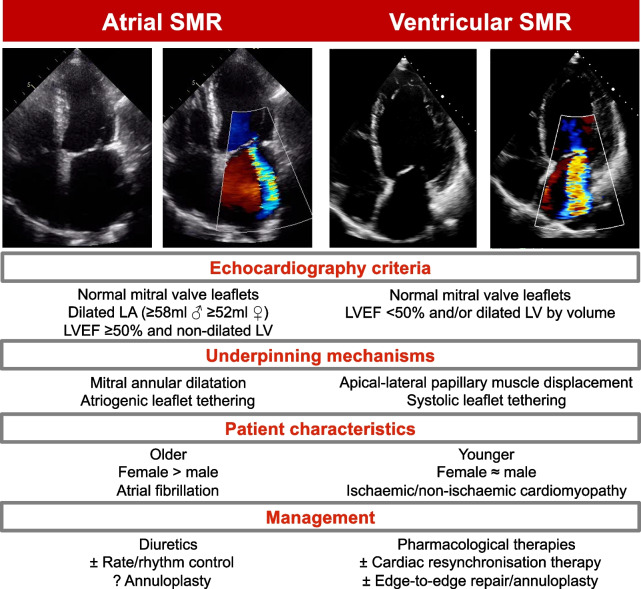

**Supplementary Information:**

The online version contains supplementary material available at 10.1186/s44156-023-00015-y.

## Highlights

- Atrial secondary mitral regurgitation is highly prevalent amongst patients with at least moderate-severe mitral regurgitation.
- Patients with atrial secondary mitral regurgitation are older, more likely to be female and usually have atrial fibrillation.
- Outcomes were poor, but much of this was related to advanced age and co-morbidities.
- Optimal treatments for atrial secondary mitral regurgitation are unknown but should be tailored to the distinct characteristics of this population.

**Supplementary Information:**

The online version contains supplementary material available at 10.1186/s44156-023-00015-y.

## Introduction

### Background

Mitral regurgitation (MR) is a prevalent heart valve disorder, usually classified into two broad categories; either ‘primary’ or ‘secondary’ [1]. Primary MR results from valve apparatus pathology, for example leaflet retraction, or chordae tendineae pathology, whilst secondary MR (SMR) occurs as the result of cardiac structural changes in the absence of significant leaflet pathology, and is most commonly seen in the setting of left ventricular (LV) dilatation and systolic dysfunction. In ventricular secondary mitral regurgitation (VSMR), LV remodelling results in apical-lateral displacement of the papillary muscles causing systolic tethering of the mitral leaflets and failure of morphologically normal leaflets to coapt [2]. However, an increasingly recognised number of patients develop severe MR in the absence of either valve leaflet pathology or LV systolic dysfunction in whom annular dilatation and failure of coaptation is the result of left atrial (LA) remodelling, often associated with atrial fibrillation [3]. Whilst annular dilatation can also contribute to VSMR, atrial secondary mitral regurgitation (ASMR) has been proposed as a distinct clinical entity with a unique pathophysiology. The prevalence, clinical characteristics, management, and long-term outcomes of these patients have not been well described.

### Objectives

We aimed to firstly report the prevalence of ASMR in an unselected population with grade III/IV (moderate-severe or severe) MR and secondly, to report and contrast the clinical and echocardiographic characteristics compared to other subtypes of MR. Finally, we sought to describe the long-term outcomes of patients with ASMR compared with other subtypes.

## Methods

### Study design

We performed a retrospective, observational study to explore the prevalence, characteristics and outcomes of consecutive patients who had grade III/IV MR determined by transthoracic echocardiography at a tertiary cardiology centre. Patients aged ≥ 18 years assessed between 01 January 2012 and 31 December 2013, who had MR graded as III/IV were eligible and identified by searches of the local echocardiography databases using ‘mitral regurgitation’ as the primary search term, and manually evaluated for inclusion. The institutional review board approved the study (IRB #9434 30/07/2021), and in view of the retrospective nature, individual patient consent was waived as appropriate data protection safeguards were in place.

### Data sources, definitions and outcomes

Transthoracic echocardiography images were analysed offline using Medcon (McKesson, Texas, USA). We recorded LA and LV dimensions, volumes and determined left ventricular ejection fraction (LVEF) by Simpson’s biplane method. Mitral annulus dimensions were measured using apical two-chamber, three-chamber and four-chamber imaging planes. Mitral annular area was calculated using the formula: mitral annulus area = 3.14 × commissure-commissure dimension x anteroposterior dimension / 4, as previously published [4, 5]. Mitral regurgitation was graded utilising an integrated approach according to guideline recommendations [6]. For the purposes of analysis, the aetiology of MR was grouped as being either primary (due to degenerative mitral valve disease), VSMR (due to LV dilatation/dysfunction), ASMR (due to atrial dilatation) or other (rheumatic mitral valve disease, infective endocarditis or unclassified). Classification as ASMR required grade III/IV MR, morphologically grossly normal mitral valve leaflets without evidence of prolapse or stenosis, LA dilatation, LV volume within normal limits and LVEF ≥ 50% with no regional wall motion abnormalities. VSMR required grade III/IV MR, morphologically normal mitral valve leaflets, LV dilatation and/or LVEF < 50%, with or without regional wall motion abnormalities, with or without co-existent atrial dilatation.

Clinical data and outcomes were obtained from local electronic care records and linked Office of National Statistics mortality data. Demographic and clinical data included age, sex and co-morbidities, which were hypertension, atrial fibrillation, diabetes mellitus, ischaemic heart disease and cerebrovascular disease. We recorded hospitalisations, surgical or transcatheter mitral valve interventions, and all-cause mortality. All non-elective hospital admissions prior to death or study censorship were classified as either due to or not due to heart failure, defined as the new onset of worsening of signs and symptoms of heart failure with evidence of congestion, with or without the use of intravenous diuretics. Patients were followed up until death with final censorship occurring September 2021.

### Statistical analysis

All statistical analyses were done using IBM SPSS Statistics version 26 (IBM Corporation, Armonk, NY). Normality of distribution was explored visually by distribution plots and confirmed using skewness tests. Continuous variables are presented as mean ± standard deviation if normally distributed, as median (interquartile range) if non-normally distributed and discrete variables are presented as number (percentage). Groups were compared using t-tests or one way analysis of covariance for normally distributed continuous data, Mann-Whitey or Kruskal–Wallis H tests for non-normally distributed data, and two-sided Pearson χ^2^ for categorical variables. Kaplan–Meier analysis was used to plot survival and groups compared using log-rank test. Univariate logistic regression used Cox proportional hazards, for which non-normally distributed data were log10 transformed. We also did sensitivity analyses comparing patients with ASMR and VSMR who did not undergo mitral valve intervention during follow-up. All tests were two-sided, statistical significance was defined as *p* < 0.05 and no imputation for missing data was made.

## Results

### Patients

During the study period a total of 2445 imaging datasets were identified by searches of echocardiography databases at our institution. Following manual evaluation and exclusion of serial imaging (repeated) datasets, a total of 388 individuals were identified who had at least grade III/IV MR. According to our criteria, 37 (9.5%) patients were classified as having ASMR, 113 (29.1%) as VSMR, 193 as primary MR (49.7%), whilst 45 (11.6%) were classified as having other aetiologies of mitral valve disease.

### Clinical and echocardiographic characteristics

Descriptive data contrasting patients according to aetiology of MR are displayed in Table 1. The median age was 75 (65–83) years and 197 (50.8%) were female. The most common co-morbidities were atrial fibrillation (44.4%), ischaemic heart disease (35.8%) and hypertension (29.6%). Compared to MR of other subtypes, patients with ASMR were on average older (median age 82 [74–87] years, *p* < 0.001), were more likely to be female (67.6%, *p* = 0.004) and more frequently had atrial fibrillation (83.8%, *p* = 0.001). Ischaemic heart disease was more common in patients with VSMR (59.5%, *p* < 0.001), whilst the distribution of diabetes mellitus and hypertension were similar.

Echocardiography data contrasting patients according to the type of mitral regurgitation are displayed in Table 1. Those with VSMR had more dilated and impaired LV systolic function. The distribution of LA volumes was similar between groups. Mitral annulus dimensions and area were greatest in patients with VSMR, but similar between patients with ASMR and primary MR. Those with primary (75.1%) or other (64.4%) aetiologies of MR were most likely to have 4 + MR, whilst those with ASMR were most likely to have moderate/severe tricuspid regurgitation.

### Mitral valve interventions

Overall, 76 (19.6%) patients underwent mitral valve intervention following a median of 270 (71–549) days, of which 39 (10.1%) were surgical mitral valve replacements and 37 (9.5%) were mitral valve repairs. No patients underwent percutaneous mitral valve intervention during the study period. Intervention for mitral valve disease was less commonly undertaken for ASMR compared to other aetiologies of mitral valve disease. Three patients underwent surgical mitral valve repair (annuloplasty ring), with concomitant tricuspid annuloplasty ring (n = 1) surgical atrial fibrillation ablation (n = 1) or both (n = 1). Patients with other aetiologies of MR, including rheumatic valve disease and infective endocarditis were the most likely to undergo mitral valve intervention (35.6%, *p* = 0.001) and mitral valve replacement (28.9%, *p* < 0.001). The distribution of mitral valve interventions and additional procedures undertaken at the time of operation are displayed in Additional file 1: Table S1.

### Outcomes

**Fig. 1:**
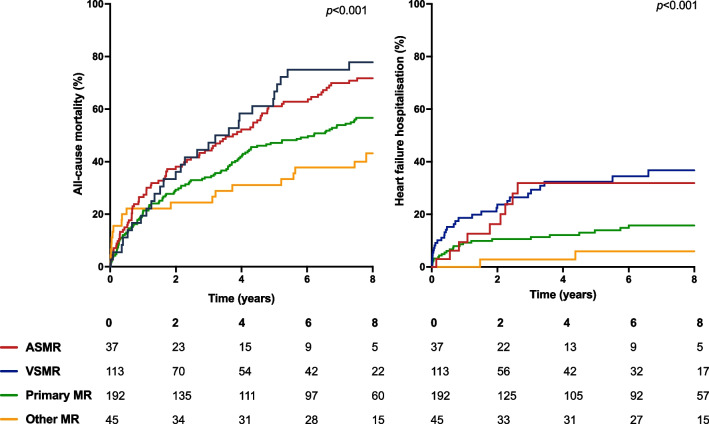
Kaplan–Meier plots of all-cause mortality and heart failure hospitalisation divided by aetiology of mitral regurgitation

During a mean follow-up of 4.7 ± 3.3 years a total of 245 (63.1%) patients died. Unadjusted all-cause mortality was highest in patients with ASMR, and lowest in those with primary or other aetiologies of MR (*p* < 0.001) (Fig. 1). Accounting for differences in age and sex between groups, using adjusted Cox proportional hazards regression, survival was not different between patients with ASMR or VSMR (hazard ratio [HR] 0.81, 95% confidence interval [CI] 0.52–1.25), which was also the case in a sensitivity analysis restricted to those who did not undergo mitral valve intervention (HR 1.12, 95% CI 0.70–1.79).

In total 69 (17.8%) patients were hospitalised for worsening heart failure which was more commonly observed in patients with ASMR (24.3%) or VSMR (28.3%) than patients with primary (13.5%) or other (4.4%) aetiologies (*p* < 0.001) (Fig. 1). Comparing patients with ASMR and VSMR, there was no significant difference in the risk of heart failure hospitalisation once age and sex were accounted for (HR 0.74, 95% CI 0.34–1.58). With similar findings when the analysis was restricted to those who did not undergo mitral valve intervention (HR 0.99, 95% CI 0.43–2.27). The total number of heart failure hospitalisations was highest in patients with ASMR (*p* = 0.001) with 5 (13.5%) patients having multiple hospitalisations for heart failure.

Within the entire cohort, variables associated with all-cause mortality or hospitalisation for worsening heart failure included age, history of atrial fibrillation or ischaemic heart disease, lower LVEF, higher LA volume, the presence of other significant valvular heart disease and pulmonary and right ventricular systolic pressures (Table 2). Within patients with ASMR, the only variables associated with outcomes were age (HR 1.07 [95% CI 1.02–1.12] per year) and co-morbidities, specifically ischaemic heart disease (HR 2.38 [95% CI 1.13–5.03]) and history of cerebrovascular disease (HR 2.81 [95% CI 1.02–7.72]). In the VSMR group, aside from older age only moderate/severe aortic stenosis and right ventricular systolic pressure were associated with heart failure hospitalisation or all-cause mortality.

**Table 1:** Clinical, demographic and echocardiographic characteristics split by aetiology of mitral regurgitation

	All (n = 388)	ASMR (n = 37)	VSMR (n = 113)	Primary MR (n = 193)	Other MR (n = 45)	*p*-value
*Demographics*						
Age (years)	75 (65–83)	82 (74–87)	76 (66–82.5)	75 (65–82)	69 (49.5–78)	** < 0.001**
Female sex [n(%)]	197 (50.8)	(25 (67.6)	55 (48.7)	86 (44.6)	31 (68.9)	**0.004**
*Past medical history*						
AF [n(%)]	200 (44.4)	31 (83.8)	54 (48.2)	95 (49.5)	20 (44.4)	**0.001**
Hypertension [n(%)]	114 (29.6)	15 (40.5)	34 (30.6)	53 (27.6)	12 (26.7)	0.43
IHD [n(%)]	138 (35.8)	16 (43.2)	66 (59.5)	51 (26.6)	5 (11.1)	** < 0.001**
Diabetes [n(%)]	49 (12.7)	4 (10.8)	17 (15.3)	20 (10.4)	8 (17.8)	0.43
Stroke/TIA [n(%)]	47 (12.2)	5 (13.5)	14 (12.6)	25 (13.0)	3 (6.7)	0.69
*Left ventricle*						
LVEDd (mm)	53.0 ± 9.1	48.0 ± 8.9	57.6 ± 9.0	52.1 ± 8.2	49.3 ± 1.3	** < 0.001**
LVESd (mm)	38.0 ± 11.0	33.1 ± 7.6	46.8 ± 11.5	35.1 ± 8.6	32.2 ± 8.2	** < 0.001**
LVEDV (ml)	136 (102.8–183)	95.5 (67.8–112.8)	158 (124–205)	136 (104–182.8)	123 (100–142)	** < 0.001**
LVESV (ml)	59 (40.8–90)	36.5 (23.3–45.8)	102 (72–140)	53.5 (39–77)	52 (36–69.5)	** < 0.001**
LVEF (%)	52.8 ± 15.9	62.3 ± 7.6	37.6 ± 14.9	58.7 ± 11.9	57.9 ± 11.7	** < 0.001**
*Left atrium*						
LA diameter (mm)	46 (41–52)	47 (42–53)	47 (41.3–50.8)	47 (41–52)	45 (41–51)	0.82
LA volume (ml)	89 (69–89)	89 (74–138)	88 (69.5–115)	90 (67–131)	92.5 (68–117.8)	0.77
*Valvular dysfunction*						
MR severity 4 + [n(%)]	262 (67.5)	22 (59.5)	66 (58.4)	145 (75.1)	29 (64.4)	**0.014**
MA diameter A2c (cm)	34.9 ± 6.3	34.2 ± 5.5	36.3 ± 6.4	34.8 ± 6.4	32.5 ± 5.8	**0.006**
MA diameter A3c (cm)	32.7 ± 6.2	34.1 ± 6.5	34.2 ± 6.1	32.0 ± 5.8	31.0 ± 6.9	**0.003**
MA diameter A4c	35.5 ± 6.3	35.4 ± 7.3	36.4 ± 5.8	35.3 ± 6.3	34.1 ± 6.8	0.20
MA diameter A3c/A2c ratio	0.95 (0.83–1.06)	1.01 (0.89–1.10)	0.95 (0.85–1.07)	0.94 (0.80–1.06)	0.96 (0.80–1.07)	0.19
MAA (cm^2^)	9.1 ± 3.0	9.0 ± 3.5	9.9 ± 3.1	8.8 ± 2.8	8.1 ± 2.9	**0.002**
Moderate/severe TR [n(%)]	152 (39.2)	26 (70.3)	52 (46.0)	59 (30.9)	15 (33.3)	** < 0.001**
Moderate/severe AS [n(%)]	44 (11.3)	0 (0)	18 (15.9)	21 (10.9)	5 (11.1)	0.067
Moderate/severe AR [n(%)]	42 (10.8)	2 (5.7)	9 (8.3)	20 (10.4)	11 (25.0)	**0.014**
*Right heart*						
PASP (mmHg)	52.2 ± 17.3	56.7 ± 14.5	52.4 ± 14.1	50.9 ± 18.0	53.0 ± 24.1	0.47

Bold values indicate* p* value < 0.05

*ASMR* atrial functional mitral regurgitation, *VSMR* ventricular functional mitral regurgitation, *AF* atrial fibrillation, *IHD* ischaemic heart disease, *TIA* transient ischaemic attack, *LVEDd* left ventricular end-diastolic diameter, *LVESd* left ventricular end-systolic diameter, *LVEDV* left ventricular end-diastolic volume, *LVESV* left ventricular end-systolic volume, *LVEF* left ventricular ejection fraction, *LA* left atrium, *MR* mitral regurgitation, *MA* mitral annulus, *A2c* apical two-chamber, *A3c* apical three-chamber, *A4c* apical four-chamber, *MAA* mitral annulus area, *TR* tricuspid regurgitation, *AS* aortic stenosis, *AR* aortic regurgitation, *RVSP* right ventricular systolic pressure, *PASP* pulmonary artery systolic pressure

**Table 2:** Univariate regression analysis of all-cause mortality and hospitalisation with worsening heart failure

Variable	All patients		ASMR		VSMR	
	Hazard ratio (95% CI)	*p-*value	Hazard ratio (95% CI)	*p-*value	Hazard ratio (95% CI)	*p-*value
Age (per year)	1.05 (1.04–1.07)	** < 0.001**	1.07 (1.02–1.12)	**0.005**	1.04 (1.02–1.06)	** < 0.001**
Male sex	0.99 (0.77–1.26)	0.92	0.73 (0.33–1.61)	0.43	1.04 (0.67–1.58)	0.84
AF	1.42 (1.10–1.82)	**0.006**	2.12 (0.64–7.04)	0.22	1.09 (0.72–1.66)	0.67
Hypertension	0.85 (0.65–1.12)	0.25	0.52 (0.24–1.15)	0.11	0.87 (0.55–1.37)	0.54
IHD	2.00 (1.56–2.57)	** < 0.001**	2.38 (1.13–5.03)	**0.023**	1.51 (0.98–2.33)	0.063
Diabetes	1.36 (0.96–1.94)	0.087	2.07 (0.71–6.03)	0.18	0.94 (0.52–1.73)	0.84
Stroke/TIA	1.34 (0.95–1.89)	0.099	2.81 (1.02–7.72)	**0.046**	1.06 (0.58–1.95)	0.86
LVEDd (per mm)	0.99 (0.98–1.01)	0.43	0.97 (0.93–1.02)	0.21	0.98 (0.96–1.00)	0.11
LVESd (per mm)	1.01 (1.00–1.02)	0.081	0.99 (0.94–1.04)	0.70	0.99 (0.97–1.01)	0.32
LVEDV (per tenfold increase)	0.51 (0.25–1.05)	0.067	0.13 (0.008–2.22)	0.16	0.51 (0.16–1.65)	0.26
LVESV (per tenfold increase)	1.48 (0.92–2.39)	0.11	1.09 (0.16–7.41)	0.93	0.78 (0.34–1.75)	0.54
LVEF (per %)	0.98 (0.98–0.99)	** < 0.001**	0.95 (0.90–1.01)	0.091	1.00 (0.98–1.01)	0.76
LA diameter (per tenfold increase)	6.05 (1.40–26.1)	**0.016**	7.10 (0.33–153.5)	0.21	4.00 (0.26–62.8)	0.32
LA volume (per tenfold increase)	1.90 (1.08–3.32)	**0.025**	2.21 (0.44–11.2)	0.34	1.48 (0.51–4.26)	0.47
MR severity 4 +	0.98 (0.75–1.27)	0.87	1.12 (0.53–2.35)	0.77	0.78 (0.51–1.18)	0.24
MAA (per cm^2^)	1.01 (0.97–1.06)	0.60	0.96 (0.85–1.09)	0.54	1.02 (0.96–1.09)	0.49
Moderate/severe TR	2.14 (1.67–2.75)	** < 0.001**	1.68 (0.74–3.82)	0.21	0.18 (0.78–1.78)	0.44
Moderate/severe AS	1.85 (1.31–2.61)	**0.001**	–	–	1.86 (1.08–3.18)	**0.025**
Moderate/severe AR	1.32 (0.91–1.93)	0.14	1.74 (0.40–7.54)	0.46	1.58 (0.76–3.27)	0.22
RVSP (per tenfold increase)	5.17 (2.42–11.0)	** < 0.001**	2.06 (0.16–26.5)	0.58	6.69 (1.41–31.8)	**0.017**
PASP (per mmHg)	1.01 (1.01–1.02)	**0.001**	1.01 (0.98–1.04)	0.61	1.01 (1.00–1.03)	0.14

Bold values indicate* p* value < 0.05

*ASMR* atrial functional mitral regurgitation, *VSMR* ventricular functional mitral regurgitation, *AF* atrial fibrillation, *IHD* ischaemic heart disease, *TIA* transient ischaemic attack, *LVEDd* left ventricular end-diastolic diameter, *LVESd* left ventricular end-systolic diameter, *LVEDV* left ventricular end-diastolic volume, *LVESV* left ventricular end-systolic volume, *LVEF* left ventricular ejection fraction, *LA* left atrium, MR mitral regurgitation, *MAA* mitral annulus area, *TR* tricuspid regurgitation, *AS* aortic stenosis, *AR* aortic regurgitation, *RVSP* right ventricular systolic pressure, *PASP* pulmonary artery systolic pressure

## Discussion

SMR occurs as the result of cardiac structural or functional changes in the absence of primary valve leaflet pathology. This broad term is usually applied to MR resulting from LV dilatation and/or systolic dysfunction, in which apical-lateral displacement of papillary muscles and systolic tethering cause failure of coaptation [2]. However, SMR is increasingly recognised in the absence of these features. In ASMR, mitral annulus dilatation due to increased LA volumes coupled with insufficient leaflet remodelling and atriogenic leaflet tethering result in failure of coaptation. [7]

Our analysis reveals that ASMR was common within an unselected cohort of patients with at least moderate-severe MR, with a prevalence of 9.5%, broadly in line with previous reports [7, 8]. Consistent with our criteria, those with ASMR had higher LVEF, and smaller LV dimensions compared to those with VSMR. It was notable, however, that the distributions of LA volumes were similar, suggesting LA dilatation may also be a contributor to the development and progression of MR in many patients with VSMR. Patients with ASMR had less elliptical mitral annuluses (as evidenced by higher apical three-chamber/two-chamber diameter ratio) due to dilatation in the antero-posterior direction, although this comparison did not reach statistical significance.

Prior studies reporting the characteristics and outcomes in ASMR have used varying definitions and imaging modalities, and have been limited to comparator populations of either VSMR or primary MR. Applying a definition requiring morphological normal mitral valve leaflets, LA dilatation and normal LV dimensions and function to an unselected population with at least moderate-severe MR, we identified a phenotypically distinct group of patients. Atrial fibrillation was highly prevalent (83.8%), which may be a major driver, or indeed consequence of the underlying pathophysiology. That some individuals had no documented history of paroxysmal or persistent atrial fibrillation suggests this is not a prerequisite for the development of ASMR, rather, an atrial myopathy resulting in deformation or dilatation could underpin this process. These findings were in keeping with prior studies, which have suggested that co-morbidities, particularly atrial fibrillation, hypertension and diabetes mellitus are highly prevalent within this population [9].

In our analysis, all-cause mortality was highest in patients with ASMR, although similar to VSMR, and less favourable compared to primary or other aetiologies once age and sex were accounted for. Prior studies contrasting patients with ASMR and VSMR have suggested that the prognosis of VSMR is less favourable, with differential predictors of outcomes between these distinct groups. This may in part be explained by differing definitions applied across studies, with those with SMR in the current study being classified as having VSMR if they had any degree of LV systolic dysfunction or dilatation [10, 11]. Patients with ASMR had a median age of 82 [74–84] years, and although the age range included patients aged 61–96 years, suggesting it does not solely affect older populations, prognosis in ASMR was most closely related to advanced age and co-morbidities. In VSMR, outcomes were primarily related to the presence of ischaemic heart disease and aortic stenosis. In other studies it has been suggested that LV dilatation and more severe MR are associated with worse prognosis [12], but this was not observed in our data. Heart failure hospitalisation was commonly observed in both ASMR and VSMR, and was significantly higher compared to primary or other aetiologies, and, although the total number of hospitalisations was highest for patients who had ASMR a time to event analysis was similar for ASMR and VSMR once age and sex were accounted for.

For patients with heart failure with reduced ejection fraction, disease modifying pharmacological therapies and cardiac resynchronisation therapy are associated with reductions in LV volumes and the severity of SMR [13]. Persistent MR despite optimised pharmacological and device therapies in the presence of LV systolic dysfunction is associated with worse outcomes [14]. For these patients, surgery to correct valvular dysfunction has not proven to be successful, with two randomised controlled trials of surgical annuloplasty ring in addition to coronary artery bypass grafting [15] and mitral valve repair or chordal-sparing replacement [16] failing to demonstrate improvements in outcomes—perhaps because these interventions target the valvular apparatus, rather than the underlying pathophysiology. More recently, two trials of transcatheter mitral valve repair using the Mitra-Clip (Abbott) device in patients with LV systolic dysfunction and moderate-severe [17] or severe [18] MR reached divergent results, leading to the plausible (but unproven) concept of proportionate and disproportionate SMR [19]. Despite this, there are now guideline recommendations that leaflet treatments for VSMR can be considered in selected patients [20]. Meanwhile, the landscape is advancing rapidly, with several percutaneous annular treatments under investigation, including a multi-centre blinded, sham-controlled study powered for patient-orientated clinical outcomes [21].

In contrast, LA remodelling resulting in secondary MR cannot currently be directly targeted by pharmacological therapies, with management limited to diuretics and rate or rhythm control of atrial fibrillation. Electrical cardioversion has been shown to restore mitral annular dynamics [22], whilst maintenance of sinus rhythm following ablation is associated with a reduction in LA volumes [23] with both approaches associated with a improvements in the degree of ASMR. Few of our patients underwent mitral valve surgery and none underwent transcatheter mitral valve intervention as our cohort predates its widespread availability and although technically feasible, these devices have not been established in this setting. Given that ASMR requires annular dilatation, an annuloplasty device might be a more logical choice. The Carillon (Cardiac Dimensions) mitral valve annuloplasty system reduces annular dimensions and MR in patients with severe [24] and non-severe [25] secondary MR who have LV systolic dysfunction and, despite the absence of randomised trials, is approved for treating ASMR in several regions. Regardless of the approach taken, the average age and burden of co-morbidities in this population should be taken into account when designing future trials.

### Limitations

This was a retrospective study, conducted in a single centre and our findings should be interpreted considering this. Three-dimensional transthoracic echocardiography or transoesophageal echocardiography imaging data were not available for all patients, which may have helped further refine classification, although two-dimensional echocardiography for SMR likely represents usual care in many settings. We do not report objective measures of mitral regurgitation severity, however our aim was to contrast the characteristics, management and outcomes of these groups diagnosed according to an integrated approach as part of usual care. Patients received intervention for mitral valve disease at the discretion of their physician or surgeon, and the cohort predated the widespread availability of transcatheter or transvenous mitral valve interventions. Due to a small number of events within each group, multivariable analyses were not undertaken.

### Conclusion

Our data suggest that ASMR represents a prevalent disease process associated with a distinct clinical phenotype and a poor prognosis. Much of the increased risk of death or heart failure hospitalisation was related to age and co-morbidities, such that therapeutic strategies for ASMR are urgently required to prove their benefit and should be tailored to the distinct characteristics of this population.

## Supplementary Information

**Additional file 1: Table S1. **Mitral valve procedures divided by aetiology of mitral regurgitation.

## Abbreviations

ASMR: Atrial secondary mitral regurgitation
CI: Confidence interval
HR: Hazard ratio
LA: Left atrial/atrium
LV: Left ventricular/ventricle
LVEF: Left ventricular ejection fraction
MR: Mitral regurgitation
SMR: Secondary mitral regurgitation
VSMR: Ventricular secondary mitral regurgitation
