# Supplementary material for: Atrial secondary mitral regurgitation: prevalence, characteristics, management, and long-term outcomes
Source: Echo Res Pract. 2023 Mar 8;10:4. doi: 10.1186/s44156-023-00015-y (PMC9993529; doi:10.1186/s44156-023-00015-y)
Supplement: Supplementary file 1 — Additional file 1: Table S1. Mitral valve procedures divided by aetiology of mitral regurgitation. [file 44156_2023_15_MOESM1_ESM.docx]

Table S1. Mitral valve procedures split by aetiology of mitral regurgitation.

|  | **All (n=388)** | **ASMR (n=37)** | **VSMR (n=113)** | **Primary MR (n=193)** | **Other MR (n=45)** | ***p*-value** |  |
| --- | --- | --- | --- | --- | --- | --- | --- |
| Mitral valve intervention | | | | | | | |
| Any mitral valve procedure [n(%)] | 76 (19.6) | 3 (8.1) | 29 (25.7) | 28 (14.6) | 16 (35.6) | **0.001** |  |
| Mitral valve repair [n(%)] | 37 (9.5%) | 3 (8.1%) | 16 (14.2%) | 15 (7.8%) | 3 (6.7%) | 0.26 |  |
| Mitral valve replacement [n(%)] | 39 (10.1) | 0 (0) | 13 (11.5) | 13 (6.7) | 13 (28.9) | **<0.001** |  |
| Other procedure during mitral intervention | | | | | | | |
| Aortic valve surgery [n(%)] | 12 (3.1) | 0 (0) | 2 (1.8) | 6 (3.1) | 4 (8.9) | 0.076 |  |
| Tricuspid valve surgery [n(%)] | 15 (3.9) | 2 (5.4) | 5 (4.4) | 4 (2.1) | 4 (8.9) | 0.17 |  |
| CABG [n(%)] | 12 (3.1) | 0 (0) | 6 (5.3) | 5 (2.6) | 1 (2.2) | 0.35 |  |
| AF ablation [n(%)] | 19 (4.9) | 2 (5.4) | 6 (5.3) | 9 (4.7) | 2 (4.4) | 0.99 |  |
| PFO closure [n(%)] | 1 (0.3) | 0 (0) | 1 (0.9) | 0 (0) | 0 (0) | 0.49 |  |
| LAA closure [n(%)] | 5 (1.3) | 0 (0) | 3 (2.7) | 2 (1.0) | 0 (0) | 0.42 |  |

ASMR; atrial functional mitral regurgitation, VSMR; ventricular functional mitral regurgitation, CABG; coronary artery bypass grafting, LAA; left atrial appendage, DCCV; direct current cardioversion.
